# Efficient In Vivo Electroporation of the Postnatal Rodent Forebrain

**DOI:** 10.1371/journal.pone.0001883

**Published:** 2008-04-02

**Authors:** Camille Boutin, Simone Diestel, Angélique Desoeuvre, Marie-Catherine Tiveron, Harold Cremer

**Affiliations:** IBDML, CNRS/Université de Méditerranée, Campus de Luminy, Marseille, France; Université Pierre et Marie Curie, France

## Abstract

Functional gene analysis in vivo represents still a major challenge in biomedical research. Here we present a new method for the efficient introduction of nucleic acids into the postnatal mouse forebrain. We show that intraventricular injection of DNA followed by electroporation induces strong expression of transgenes in radial glia, neuronal precursors and neurons of the olfactory system. We present two proof-of-principle experiments to validate our approach. First, we show that expression of a human isoform of the neural cell adhesion molecule (hNCAM-140) in radial glia cells induces their differentiation into cells showing a neural precursor phenotype. Second, we demonstrate that p21 acts as a cell cycle inhibitor for postnatal neural stem cells. This approach will represent an important tool for future studies of postnatal neurogenesis and of neural development in general.

## Introduction

The increasing use of high throughput gene expression studies, that generate enormous amounts of data in a single experiment, renders classical techniques like transgenesis via oocyte injection or gene knockout by homologous recombination in embryonic stem cells often too time intensive and costly for systematic functional screening of candidate genes. Therefore, the development of alternative methods represents a priority.

Gene transfer through injection of DNA or RNA into embryos followed by electric pulses has developed into an important tool for functional analyses in vivo. This has, for example, been achieved by *in ovo* electroporation in the chick [1] or by *in utero* electroporation in rodents [2]. Both these techniques are widely used and of outstanding importance for functional studies [3]. More recently gene transfer in the postnatal retina [4], [5] and the cerebellum [6] have been demonstrated.

In the postnatal and adult forebrain neural stem cells lining the lateral ventricle (LV) generate transit amplifying progenitors that produce migratory neuronal precursors. After long distance migration in the rostral migratory stream (RMS) these precursors differentiate into periglomerular and granule interneurons neurons of the olfactory bulb (OB) [7]. We show here that radial glia cells, the neural stem cells in the postnatal SVZ, can be efficiently electroporated. Over the subsequent days the entire neurogenic system and the differentiated interneurons in the OB express the introduced transgenes strongly, allowing efficient manipulation of the different cell types. We performed two proof-of-principle experiments to demonstrate the usefulness of this approach for functional gene analyses. First, we show that expression of the Neural Cell Adhesion Molecule (NCAM) in neural stem cells strongly interferes with radial glia maintenance and induces the generation of migratory neuronal precursors. Second, we overexpressed the cell cycle inhibitor p21 and demonstrate its capacity to interfere with the proliferation of neural stem cells.

## Results

**Figure 1:**
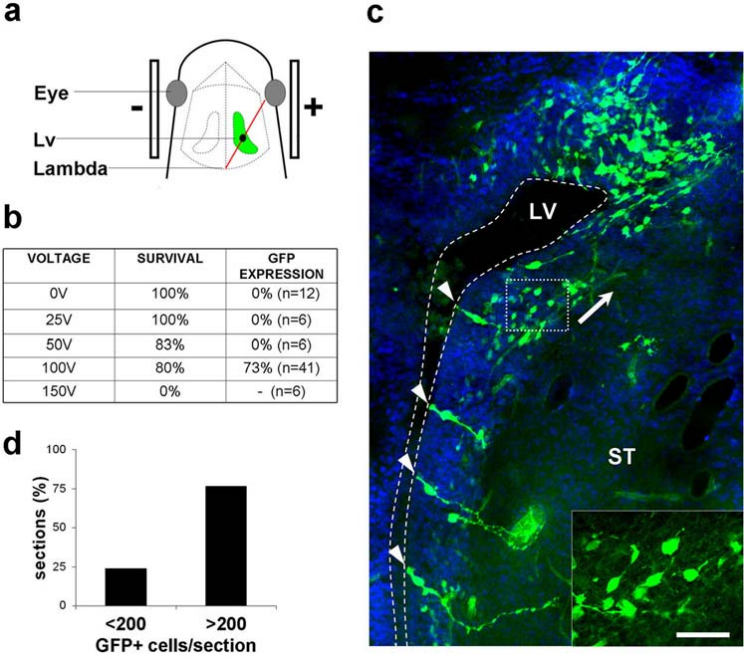
Electroporation of the postnatal forebrain. (a) A virtual line (red) connecting the right eye to the craniometrical landmark lambda served as positional marker for DNA injection. The incision point is indicated as a dot. Lateral bars indicate the position of the electrodes. (b) Table recapitulating the tested voltages, survival after electric shocks and the success rate concerning GFP-expression. (c) Example of a coronal section through a GFP-electroporated forebrain at the level of the lateral ventricle (LV) at 2dpe. A section containing relatively few positive cells was selected to simplify identification of the different cell types. The section was counterstained with Hoechst 33258 to facilitate orientation. Strongly GFP positive cells with the morphology of radial glia are visible in the ventricular zone (arrowheads), while cells with generally lower levels of GFP expression are organized mainly parallel to the ventricular surface (see high magnification of the boxed area in the insert). Direction of processes is suggestive of migration towards the dorso-lateral edge of the ventricle (arrow). (d) Evaluation of electroporation efficiency. Histological sections were grouped in bins representing sections containing more or less than 200 cells. 75.8% of the sections were classed in the higher group. ST: striatum. Scale bar: 40 µm; 20 µm in the insert.

For intraventricular injections of the right LV of neonate to four days old pups (P0-P4), a virtual line connecting the right eye with the craniometric landmark lambda was used and a small incision was positioned slightly caudal of the midpoint of this line (Fig. 1a). Subsequently, plasmid solution was injected via a pulled out glass injection capillary connected to a Hamilton syringe. Depth of injection was controlled using a stereotaxic rig. An injection was considered correct when the shape of the now darker stained lateral ventricle was visible under a strong light source. Successfully injected animals were subjected to electrical pulses using the CUY21 edit device and tweezer electrodes coated with conductive gel. Electroporated animals were reanimated and returned to the mother.

To establish the conditions, a Green Fluorescent Protein (GFP) eukaryotic expression vector based on the chicken β-actin promoter and the CMV enhancer (pCX-EGFP-N1; [8]) was used. Hypothermia in combination with electric pulses of 50V or 100V induced a low degree of lethality while pulses of 150V were lethal for all 6 animals tested (Fig. 1b). While at voltages below 100V no GFP-expressing cells could be observed, over 70% of the animals that received pulses of 100V showed varying amounts of electroporated cells along the lateral wall of the right LV (Fig. 1c). To get an estimation of the amount of trangene expressing cells over eighty GFP positive sections taken from six different animals sacrificed 2 days post electroporation (dpe) were quantitatively evaluated. These analyses showed that 75% of the sections contained more than 200 GFP expressing cells (Fig. 1d, example in Fig.S1).

Interestingly, the injection and electroporation processes had no obvious consequences on behaviour of surviving pups. After warming, the animals started immediately sucking and were indistinguishable from non-manipulated littermates within 15 min. The only obvious consequence of the electroporation process to brain morphology was a moderate extension of the right LV in about 50% of the animals analyzed (example shown in Fig.S2). TUNEL staining for the presence of apoptotic cells and DAPI staining to identify pyknotic nuclei did not reveal negative consequences of the electroporation process in all brain areas observed (data not shown).

**Figure 2:**
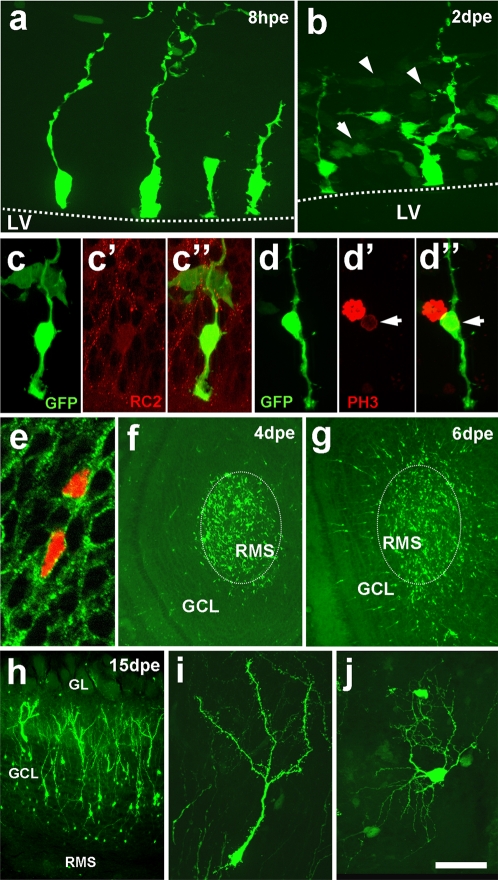
Characterization of the electroporated cells and their offspring. (a) Eight hours post electroporation (8hpe), cells with the typical morphology of radial glia exhibit strong GFP expression in the somata and processes. (b) At 2dpe radial glia cells are generally surrounded by cells showing weaker and variable GFP expression. These have no contact with the LV and show a generally tangential orientation (arrowheads). (c) Labelling of GFP positive cells in the ventricular zone (c) with anti-RC2 antibody (c') verifies their radial glia identity (merged image in c”). Mark that weaker GFP expressing neuronal precursors are not labelled for RC2, as expected. (d) a sub-population of GFP+ radial glia cell (d) expresses the mitotic marker PH3 (d'; 1dpe), merge in (d”) and are therefore proliferating. (e) Nuclear RFP-expression (in red) in combination with PSA-NCAM staining (green, antibody MenB) in the RMS at 4dpe identifies offspring of the electroporated cells as migratory neuronal precursors. (f) At 4dpe large numbers of GFP expressing cells arrive in the OB via tangential migration in the RMS. (g) At 6dpe GFP positive cells switch to radial migration and invade the granule cell layer (GCL). (h) After 15dpe large amounts of GFP expressing cells with complex morphologies can be identified in the OB. Higher magnification shows that these cells have neuronal morphology of the granule (i) and periglomerular (j) type. RMS: rostral migratory stream; GL: glomerular layer; LV: lateral ventricle. Scale bar: 20 µm in a,b,e; 15 µm in c,d; 300 µm in f,g; 100 µm in g; 30 µm in i,j.

We characterized the electroporated cells and their offspring. Until 8 hours post electroporation, only cells bordering the wall of the LV showed GFP expression (Fig. 2a). Evaluation of 216 individual GFP positive cells (4 mice) at high magnification revealed typical radial glia morphology, namely an apical process in contact with the LV and a thin basal fiber that often extended to the pial surface. Only 12 of 216 cells could not be doubtlessly categorized as radial glia. Immunohistochemical labelling using RC2 [9] further confirmed the radial glia identity of the transfected cells (Fig. 2c-c”). In addition, a subfraction of the GFP+ radial glia cells expressed the mitotic marker Phosphohistone H3 (Fig. 2d-d”) suggesting that actively proliferating cells can be targeted.

Two days after electroporation most radial glia cells were surrounded by clusters of cells that showed lower and varying degrees of GFP expression (Fig. 1c, 2b). In general, these cells had a spindle like morphology, no contact to the LV and were aligned parallel to the ventricular surface (insert in Fig. 1c, arrowheads in Fig. 2b). They were oriented mainly towards the dorso-lateral edge of the LV, where large numbers of GFP+ cells accumulated (Fig. 1c). Electroporation of an expression-vector encoding the Red Fluorescent Protein carrying to a nuclear localization signal (Histone2B-mRFP; [8]) in combination with immunostaining for PSA-NCAM (Fig. 2e) or doublecortin (not shown) identified this population as migratory neuronal precursors.

Four days after electroporation large numbers of GFP+ cells were visible along the entire RMS (not shown) and in the centre of the OB (Fig. 2f). At 6dpe radial migration of GFP+ precursors away from the RMS and towards the granule and periglomerular layers of the OB became apparent (Fig. 2g).

Fifteen days after electroporation, the latest time point observed, GFP+ cells with radial glia morphology became sparse while the OB contained a substantial number of GFP+ cells (Fig. 2h). High magnification analysis revealed the typical morphology and position of granule (Fig. 2i) and periglomerular (Fig. 2j) neurons of the OB.

To test the efficiency of co-electroporation of different vectors in our system, we electroporated varying concentrations of GFP and RFP expression plasmids and identified single or double positive cells at 2dpe (Fig.S3). Quantitative analysis revealed that at a ratio of 3∶1, 85.4% of the GFP+ cells co-expressed RFP.

**Figure 3:**
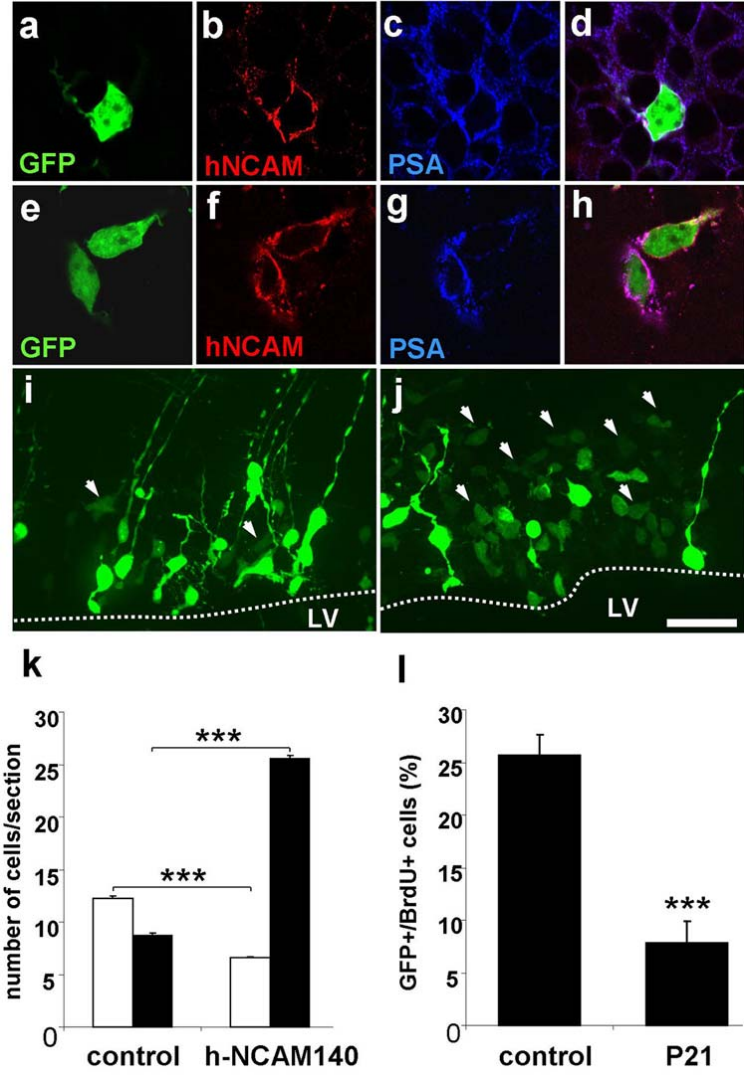
Expression of hNCAM140 and p21 in the postnatal forebrain. (a–h) Expression of human NCAM 140 in wt (a–d) and NCAM deficient (e–h) mice. Co-electroporation of GFP (a) with a hNCAM-140 expression plasmid (b, hNCAM recognized by specific antibody 123C3) in the wildtype context led to slightly enhanced PSA expression in the PSA positive SVZ (c). In contrast, hNCAM140 expression in the NCAM-deficient context (f) induced specific expression of PSA (g, merge in h) in the transfected cells. (i–k) Phenotypic consequences of hNCAM140 expression in wt mice. Co-electroporation of hNCAM140 and GFP (j) induced an increase in cells with the characteristics of migratory neuronal precursors (arrowheads) in relation to radial glia cells. (k) Quantification, open bars: radial glia; black bars: neuronal precursors – control: radial glia 12.23 +/− 0.22 cells per section, neuronal precursors 8.8 +/− 0.24 cells per section, at least 3 sections per animal, n = 6; hNCAM140: radial glia 6.59 +/− 0.06 cells per section, p<0.0001, neuronal precursors 25.53+/− 0.36 cells per section, p<0.0001, at least 3 sections per animal, n = 7). (l) Overexpression of p21 via postnatal electroporation induced a significant reduction of proliferation in the affected cells as measured by BrdU incorporation [12] (control: 25.66 +/− 2.02%, at least 4 sections per animal, n = 7; p21: 7.79 +/− 2.15%, at least 4 sections per animal, n = 5; p<0.001). Scale bar: 10 µm a–h, 30 µm in i,j.

Two paradigms have been chosen to validate the approach. First, we introduced a defined human isoform of the Neural Cell Adhesion Molecule (hNCAM140) into wildtype and NCAM-deficient mice [10]. The polysialylated form of NCAM is a widely used marker for migratory neuronal precursors [11], [12]. However, it is not expressed in neural stem cells or transit amplifying precursors surrounding the lateral ventricle [13]. Co-electroporation of an expression plasmid encoding hNCAM140 (pCX-hNCAM140, [14] and GFP in wildtype mice induced strong expression of the proteins in neuronal precursors at 2dpe (Fig. 3a–d). Polysialic acid was present on the electroporated GFP+ cells as well as on the surrounding (mouse NCAM expressing) cells (Fig 3c, overlay in d). When hNCAM140 was expressed in mutants (Fig. 3e–h) the electroporated cells were PSA positive, in a negative environment (Fig. 3g), demonstrating that the human NCAM-isoform is efficiently polysialylated. A striking phenotypic consequence of co-electroporation of hNCAM140 and GFP in wt mice was a loss of cells with radial glia morphology and an increased appearance of cells that showed the properties and localization of migratory neuronal precursors (Fig. 3i, j). Quantification of this effect demonstrated that in the control situation about equivalent numbers of both cell types were present, while when NCAM was co-electroporated there were almost four times more precursors than radial glia per section (Fig. 3k). Thus, premature expression of hNCAM in radial glia cells interferes with maintenance of this cell-type and induces the overrepresentation of cells with neuronal precursor phenotype. This result is in agreement with the finding that retroviral transduction of hippocampal progenitors with NCAM140 promotes a shift towards the neuronal phenotype [15].

As a second means to validate the electroporation approach we overexpressed the cell cycle inhibitor p21. When GFP alone was electroporated 25.06 +/− 2.02% (at least 4 sections/mouse, n = 7) of all fluorescent cells in the SVZ at 2dpe were proliferating as indicated by the incorporation of BrdU. When a p21-GFP fusion protein was expressed (plasmid pCX-p21-EGFP-N1), this value showed a significant decrease to 7.79 +/− 2.15 (at least 4 sections/mouse, n = 5). Therefore, expression of p21 in neural stem cells via postnatal electroporation inhibits their proliferation. This is in agreement with previous work, showing that loss of p21 interferes with the relative quiescence of forebrain stem cells [16].

## Discussion

Taking advantage of the continuous neurogenesis that provides during postnatal and adult stages new neurons for the OB, we developed a new electroporation method that allows the genetic alteration of radial glia, neuronal precursors and neurons *in vivo*. This approach is based on the direct manipulation of newborn mice and does not implicate complex surgical intervention on the pregnant female as in the case of *in utero* electroporation [2]. Anaesthesia by severe hypothermia induces almost no lethality, the injection implicates only a small incision of the skin and skull, positioning of the injection needle is controlled by a stereotaxtic device and the volume of the injected liquid is well defined via a microsyringe. In our hands the reliable electroporation of up to 30 pups over a two hour experiment is feasible for an experienced manipulator.

Postnatal electroporation works with comparably simple eukaryotic expression vectors that can be generated and injected in a standard laboratory without particular precautions, as in the case of virus based vectors. Multiple independent transgenes can be easily introduced in the same cell and cell type specific promoters can be used to target different cell populations. Despite the fact that expression of trangenes should be transitory, we found strongly GFP+ cells up to 15 dpe, suggesting that considerably longer time windows can be analyzed. However, for long-term studies the presence of the trangenically expressed protein should be verified. Finally, the method is theoretically suited for the direct electroporation of RNA, which is of particular importance regarding the increasing use of RNAi based knockdown approaches [17].

We validated the usefulness of postnatal electroporation using two different paradigms. First, we show that a human NCAM isoform can be efficiently introduced into the forebrain and is correctly expressed and polysialylated. A result of this gain-of-function is a shift in the ratio between radial glia like cells and migratory neuronal precursors, suggesting that premature expression of NCAM in neural stem cells induces the generation of young neurons destined for the OB. This is in agreement the finding that retroviral transduction of hippocampal progenitors with NCAM140 promotes a shift towards the neuronal phenotype [15]. Several questions concerning the function of NCAM in this system remain open. Is the effect cell autonomous or not? Is it dependent on the presence of polysialic acid on NCAM? Does the generation of neuronal precursors pass via a transit amplifying cell-type (type C cells) [13], [18]? These and other points will be addressed in future studies.

The second approach we used to validate postnatal electroporation was the expression of the cell cycle inhibitor p21. Our finding that radial glia cells that overexpress p21 show a significantly decreased proliferation rate is in perfect agreement with previous work, showing that p21 contributes to adult neural stem cell quiescence. This might be necessary for the life-long maintenance of neural stem cell self-renewal because these may be limited to a finite number of divisions [16].

In conclusion, the new method presented here should have a wide spectrum of applications for the analysis of postnatal neurogenesis, but also to study the molecular and cellular mechanisms that underlie neural development and function in general. While expression analysis in the nervous system is today largely based on large scale approaches like microarray and Serial Analysis of Gene Expression [19], the functional analysis of this expression data represents a major bottleneck. Postnatal electroporation of the forebrain will be an important approach to bypass this problem.

## Materials and Methods

### Postnatal electroporation procedure

Animals were treated according to guidelines approved by the French ethical committee. Neonate to four days old pups (P0-P4; CD1 strain, Charles-River, Lyon, France) were anesthetised by hypothermia (4 min) and fixed to a support using band-aid. The skin and the skull overlying the lateral ventricle were opened over about 2 mm using an ophthalmic scalpel. As a general positional marker, a virtual line connecting the right eye with lambda (visualized by a strong cold light source) was used and the incision was positioned 1 mm caudal to the midpoint of this line (Fig. 1a). Subsequently, the animal was placed in a stereotaxic rig (Kopff, Germany) under a Hamilton syringe connected to a pulled out glass capillary (diameter 200 µm, GC100-15, Clark, UK) containing 2 µl of plasmid solution (5 µg/µl, in PBS containing 1% Fast Green). The syringe was placed over the incision, positioned at the level of the skull, then lowered between 2.5 mm (P0) to 3.5 mm (P4) into the lumen of the right LV and the stained DNA solution was injected. An injection was considered correct when the shape of the now slightly dark stained lateral ventricle was visible under the light source. Only successfully injected animals were subjected to five electrical pulses (50 ms, separated by 950 ms intervals) using the CUY21 edit device (Nepagene, Chiba, Japan) and 10 mm tweezer electrodes (CUY650P10, Nepagene) coated with conductive gel (Control Graphique Medical, France). Electroporated animals were reanimated for several minutes on a 37°C heating plate before being returned to the mother.

### Plasmids

Plasmids used in this study contain fluorescent reporter genes or NCAM/p21 cDNAs in a eukaryotic expression vector based on the chicken β-actin promoter and the CMV enhancer (pCX-MCS2, a derivative of pCAAGS, [8]: pCX- EGFP-N1 [8], pCX-hNCAM140 [14], pCAGH2BmRFPpA; gift from S. Tajbakhsh, Inst. Pasteur, Paris, France), pCX- P21-EGFP-N1 (M. Manceau, IBDML, Marseille, France). Plasmids were prepared by using an EndoFree Plasmid Kit (Quiagen Maxiprep Kit, cat. no. 12362).) and resuspended in PBS (5 µg/µl final concentration).

### Immunohistochemistry

For histological analysis, pups were deeply anaesthetized with an overdose of xylazin/ketamin. Perfusion was performed intracardiacally with a solution of 4% paraformaldehyde in PBS. The brain was dissected out and immersed overnight in the same fixative at 4°C. Sections were cut at 50 µm using a microtome (Microm, Walldorf, Germany). Immunohistochemistry was done on floating vibratome sections as described previously [20]. Briefly, sections were first incubated overnight at 4°C with the following antibodies: 123C3 against hNCAM (1/100, R. Michalides, Amsterdam, Netherlands), MenB against PSA (1/250, provided by G. Rougon, Marseille, France), RC2 (1/200, Developmental Studies Hybridoma Bank, Iowa, USA), PH3 (1/200, Upstate, USA) before incubation with the corresponding fluorescent labelled secondary antibody. Before mounting, cell nuclei were stained with Hoechst 33258. Optical images were taken either using a fluorescence microscope (Axioplan2, ApoTome system, Zeiss, Germany) or a laser confocal scanning microscope (LSM510, Zeiss, Germany).

### BrdU administration and detection

12 hours post-electroporation four subcutaneous injection of BrdU were performed over 24 h (Sigma, Saint-Louis, MO; 50 µg/gm body weight). Brains were collected as described and processed for BrdU staining: sections were incubated for 15 min at 37°C in 2N HCl-0.5% tritonX100 pre-heated at 37°C, rinsed three times for 8 min each in 0.1 M Borat, pH 8.5 and three time (10 min) in PBS-0.1% tritonX100. Afterwards immunohistochemistry was performed as described using a primary antibody anti-BrdU (Dakocytomation, Denmark, 1/200).

## Supporting Information

Figure S1 Example of sections containing low (<200 cells, a) and or high (>200 cells, b) numbers of GFP expressing cells bordering the LV at 2dpe. Scale bar: 200 µm
(4.54 MB TIF)

Figure S2 Extension of the right lateral ventricle (b) in comparison to the contralateral side (a) as a consequence of the injection process was the only detectable morphological alteration that could be observed. Mark the low amount of GFP positive cells on the medial side of the contralateral ventricle. In rare cases DNA diffuses through the ventricular system leading to transfection of the septal ventricular zone of the left LV after electroporation. Scale bar: 400 µm
(4.54 MB TIF)

Figure S3 Co-electroporation of two fluorescent proteins and expression of human NCAM. (a–c) Co-electroporation of GFP (a) and nuclear RFP (b) expression plasmids led to over 80% of double positive cells (c) at 2dpe. Scale bar: 40 µm
(4.54 MB TIF)
