# Supplementary figures and images for: Efficient In Vivo Electroporation of the Postnatal Rodent Forebrain
Source: PLoS One. 2008 Apr 2;3(4):e1883. doi: 10.1371/journal.pone.0001883 (PMC2270900; doi:10.1371/journal.pone.0001883)

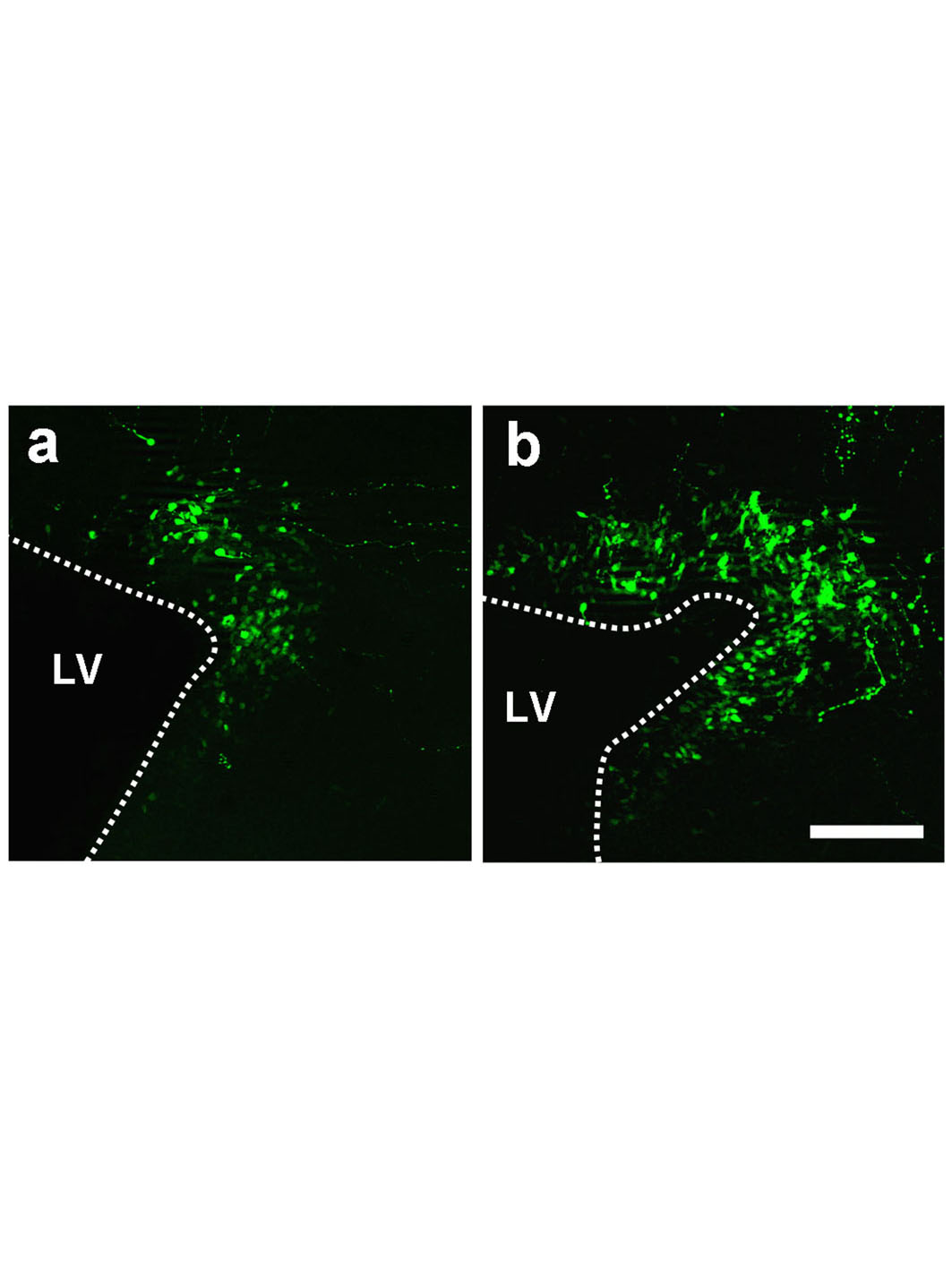

Supplement: Figure S1 — Example of sections containing low (<200 cells, a) and or high (>200 cells, b) numbers of GFP expressing cells bordering the LV at 2dpe. Scale bar: 200 µm (4.54 MB TIF) [file pone.0001883.s001.tif]

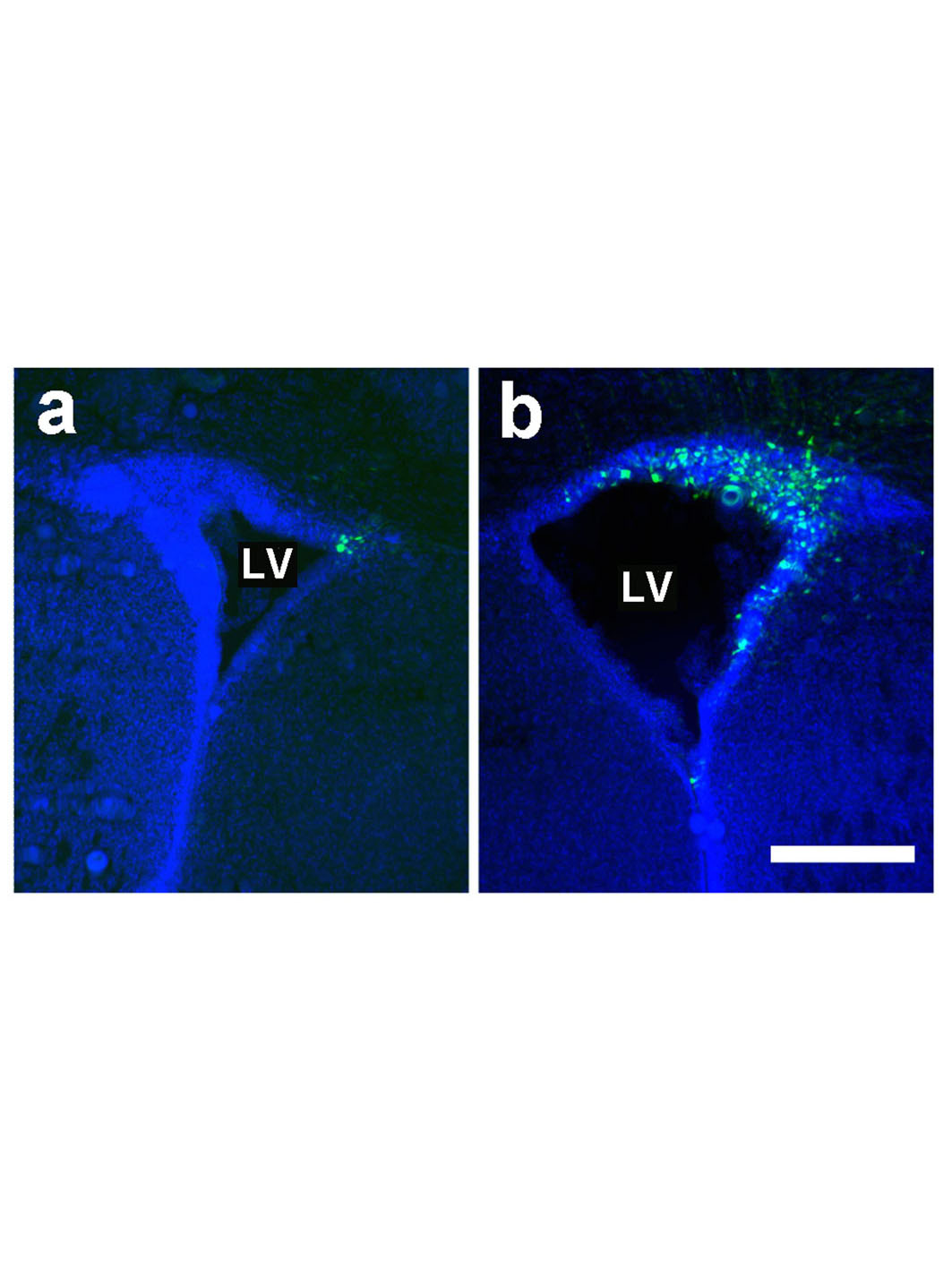

Supplement: Figure S2 — Extension of the right lateral ventricle (b) in comparison to the contralateral side (a) as a consequence of the injection process was the only detectable morphological alteration that could be observed. Mark the low amount of GFP positive cells on the medial side of the contralateral ventricle. In rare cases DNA diffuses through the ventricular system leading to transfection of the septal ventricular zone of the left LV after electroporation. Scale bar: 400 µm (4.54 MB TIF) [file pone.0001883.s002.tif]

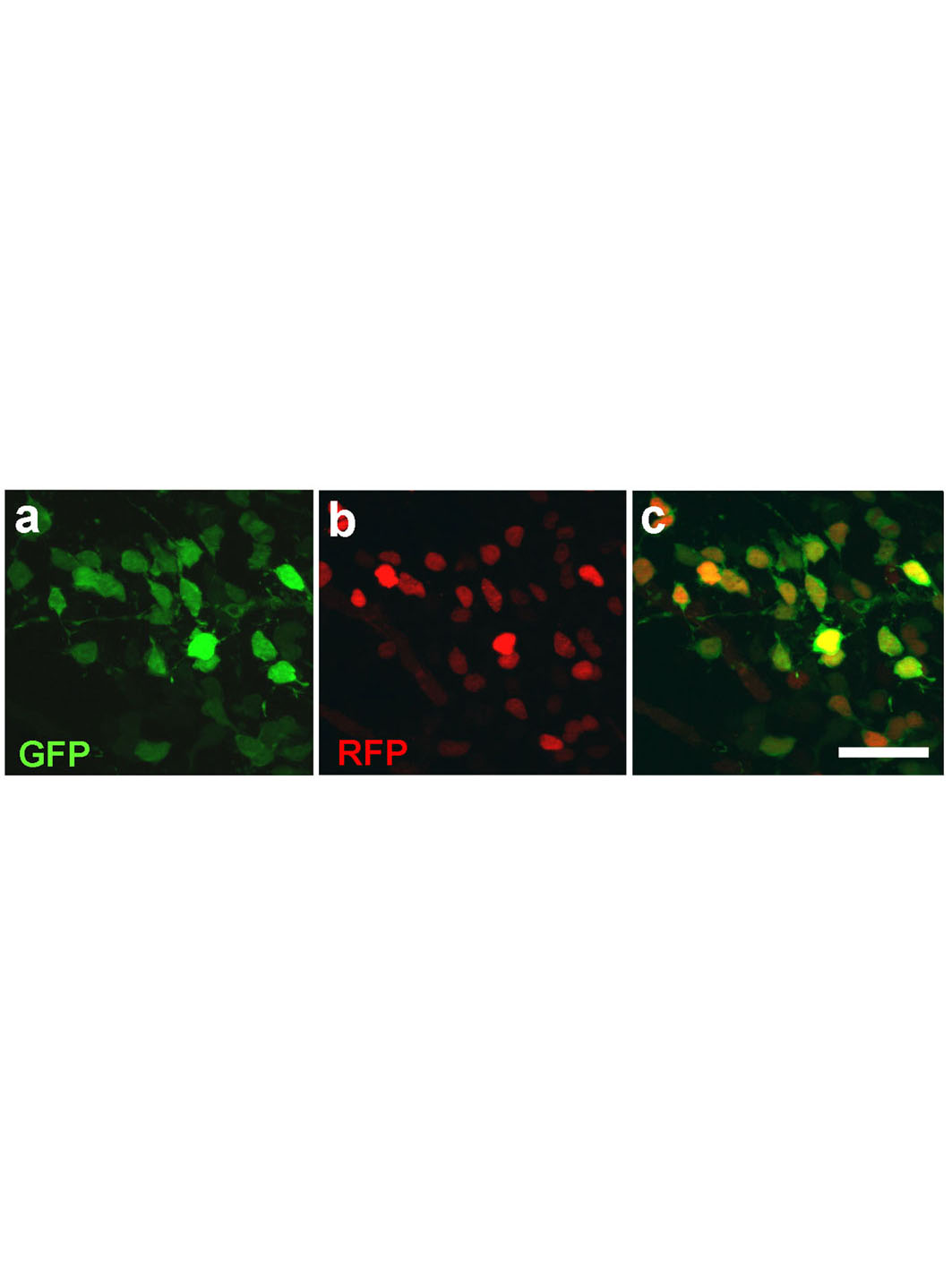

Supplement: Figure S3 — Co-electroporation of two fluorescent proteins and expression of human NCAM. (a–c) Co-electroporation of GFP (a) and nuclear RFP (b) expression plasmids led to over 80% of double positive cells (c) at 2dpe. Scale bar: 40 µm (4.54 MB TIF) [file pone.0001883.s003.tif]
